# Activity of Plazomicin (ACHN-490) against MDR clinical isolates of *Klebsiella pneumoniae, Escherichia coli*, and *Enterobacter* spp. from Athens, Greece

**DOI:** 10.1179/1973947812Y.0000000015

**Published:** 2012-08

**Authors:** Irene Galani, Maria Souli, George L Daikos, Zoi Chrysouli, Garyphalia Poulakou, Mina Psichogiou, Theofano Panagea, Athina Argyropoulou, Ioanna Stefanou, George Plakias, Helen Giamarellou, George Petrikkos

**Affiliations:** 1 4th Department of Internal Medicine, Molecular Biology Section, Athens University School of Medicine, Greece; 2 First Department of Propaedeutic Medicine, University of Athens, Greece; 3 Laboratory of Microbiology, Evaggelismos Athens General Hospital, Greece; 4 Department of Microbiology, Laikon General Hospital, Greece; 5 6th Department of Internal Medicine, Diagnostic and Therapeutic Center of Athens ‘Hygeia’, Greece

**Keywords:** Aminoglycosides, Multidrug resistant, VIM, KPC

## Abstract

The *in vitro* activity of plazomicin was evaluated against 300 multidrug resistant (MDR) (carbapenemase and/or ESBL-producing) isolates from four hospitals in Athens, an area where carbapenemase-producing organisms are endemic. Most of the isolates were also resistant to the legacy aminoglycosides with the MIC_50_/MIC_90_ to tobramycin, amikacin and gentamicin being 32/>32, 32/>32 and 4/>8 μg/ml, respectively. ACHN-490 retained activity (MICs⩽4 μg/ml) against all isolates of *Klebsiella pneumoniae, Escherichia coli*, and *Enterobacter* spp. tested with MIC_50_ and MIC_90_ of 1 and 2 μg/ml, respectively, irrespective of their MDR phenotype and it represents a promising alternative for the treatment of the most problematic Gram-negative pathogens.

## Introduction

Bacterial resistance is an increasing threat to the successful treatment of both community- and hospital-acquired infections (http://www.earss.rivm.nl) and antimicrobials potent against multidrug resistant (MDR) pathogens are urgently needed.

Plazomicin (formerly ACHN-490) (Achaogen, South San Francisco, CA, USA) is a next-generation aminoglycoside, currently in early clinical development (FDA, http://clinicaltrials.gov/), with enhanced activity against many MDR Gram-negative bacteria and *Staphylococcus aureus* including methicillin resistant S. aureus isolates (MRSA) (MIC_90_, 2 μg/ml).^1^ Plazomicin is not affected by any of known aminoglycoside-modifying enzymes, except AAC(2′)-Ia, -Ib and -Ic (only found in *Providencia spp*), it retains the favourable bactericidal properties of the aminoglycoside class and has demonstrated potent in vivo efficacy in two animal infection models.^2^ Methylation of 16S ribosomal RNA (rRNA) confers MICs of >8 μg/ml for plazomicin, as well as high-level resistance to all parenterally administered aminoglycosides that are currently in clinical use**.**^3^,^4^ The compound is currently under development for the treatment of complicated urinary tract infections and acute pyelonephritis as a single agent.

After intravenous administration of plazomicin to humans at a dose of 15 mg/kg, the maximum concentraration was 113 μg/ml, the area under the curve (0–24) was 239 hours μg/ml, the half-life was 3·0 hours and the steady-state volume of distribution was 0·24 l/kg.^5^ Human phase I and II studies to date have not reported nephrotoxicity or ototoxicity, and lack of ototoxicity has been reported in the guinea pig model.^5^

In the present work, we analyzed the *in vitro* activity of plazomicin against a collection of 300 MDR clinical isolates of *Klebsiella pneumoniae*, *Escherichia coli*, and *Enterobacter* spp. recently collected at four tertiary-care Hospitals in Athens, Greece, the University General Hospital Attikon, the Laikon and Evaggelismos General Hospitals, and the private Hospital Hygeia.

## Material and Methods

Clinical isolates collected from January 2008 to November 2010, were studied and only one isolate per patient was accepted. Identification and MIC determinations were performed using an automated system (BD Phoenix automated microbiology system; BD Diagnostic Systems, Sparks, MD, USA). MICs of plazomicin (0·25–32 μg/ml), tobramycin (0·25–32 μg/ml), and fosfomycin (16–512 μg/ml) were determined by the agar dilution method following the European Committee on Antimicrobial Susceptibility Testing (EUCAST) guidelines (www.eucast.org), whereas those of doripenem and tigecycline were determined using E-test (AB Biodisk, Solna, Sweden), in accordance with the manufacturer’s instructions. Plazomicin was supplied by Achaogen, Inc. Tobramycin and fosfomycin were purchased from Sigma-Aldrich (St Louis, MO, USA). Agar medium in which fosfomycin MICs were tested was supplemented with 25 μg/ml of glucose-6-phosphate (Sigma-Aldrich). *E. coli* ATCC 25922 and *Pseudomonas aeruginosa* ATCC 27853 were used as control strains. Results were interpreted in accordance with the EUCAST guidelines (www.eucast.org).

All isolates were screened for MBL and class A carbapenemase production with EDTA–meropenem and meropenem–boronic acid disc synergy tests,^6^ respectively. The presence of KPC and VIM genes was confirmed by PCR with specific primers.^6^ ESBL production was tested with the CLSI ESBL confirmatory test^7^ and with a modified test using clavulanate in combination with boronic acid and EDTA in Enterobacteriaceae that produced KPC or VIM enzymes.^8^

Isolates with MIC to plazomicin of 4 μg/ml were examined by PCR for the presence of 16S rRNA methylase genes (i.e., *armA*, *rmtA*, *rmtB*, *rmtC*, *rmtD*, and *npmA*) and the most common aminoglycoside-modifying enzymes in Gram-negative pathogens, using primers and conditions previously reported.^9^–^11^ In particular, the following genes were investigated: *aac(6*′*)-Ia, aac(6*′*)-Ib*, *aac(6*′*)-I*Ια**, *ant(2*′)*-Ia*, *aac(3)-Ia, aac(3)-IIa*, *aac(3)-IVα* and *aph(3*′*)-VIa*.

## Results and Discussion

The studied isolates included 241 *K. pneumoniae*, 33 *E. coli* and 26 *Enterobacter* spp*.* derived from blood (65·7%), pus (5·3%), bronchial secretions (1·7%), urine (8·0%), and fecal carriage (19·3%).

Among the *K*. *pneumoniae* isolates, 138 (57·3%) were class A carbapenemase producers; 75 (31·1%) showed a positive EDTA-meropenem disc synergy test, which was suggestive of MBL production and 14 (5·8%) were designated both class A carbapenemase and MBL producers. PCR amplification confirmed the presence of *bla*_KPC_ and *bla*_VIM_ genes in all class A carbapenemase and MBL producers, respectively. ESBL production was confirmed in 113 (81·9%) KPC producers, in 43 (57·3%) VIM producers and in 4 (28·6%) KPC and VIM producers. The remaining 14 *K*. *pneumoniae* isolates were all ESBL producers. Among the *E*. *coli* isolates, 15 (45·5%) were designated ESBL producers, whereas 9 (27·3%) were identified as KPC and 9 (27·3%) as VIM producers. Four of the VIM-positive *E*. *coli* isolates were also ESBL producers. Twenty-one (80·8%) of the *Enterobacter* spp. isolates (19 *E. cloacae* and 7 *E. aerogenes*) harboured the *bla*_VIM_ gene, while four (15·4%) harboured the *bla*_KPC_ and one isolate possessed both *bla*_VIM_ and *bla*_KPC_.

The susceptibility results for all tested antimicrobials are shown in Table 1. Of the 300 isolates tested nine were pandrug-resistant, 157 were extensively drug-resistant and the remaining 134 were MDR according to definition given by ECDC.^12^ As shown, isolates were highly resistant not only to carbapenems (MIC_50_⩾8; MIC_90_>8 μg/ml) and piperacillin-tazobactam (MIC_50_>64/4; MIC_90_>64/4 μg/ml) but also to ciprofloxacin (MIC_50_>2; MIC_90_>2 μg/ml). Approximately 78% of the strains were susceptible to colistin displaying MICs⩽2 μg/ml, while tigecycline’s MIC_50_ and MIC_90_ were 2 and 4 μg/ml, respectively (with 32% of the isolates being susceptible and 81% displaying MICs ⩽2 μg/ml). Finally, fosfomycin demonstrated 56% susceptibility with an MIC_50_ of ⩽16 and an MIC_90_ of 128 μg/ml.

**Table 1:** *In vitro* activity of plazomicin and comparators against 300 MDR enterobacterial isolates

Microorganism	Antimicrobial agent	Breakpoints (S, R)	Range (μg/ml)	MIC_50_	MIC_90_	Susceptibility rate (%)
All	Plazomicin	NA	⩽0·25 to 4	1	2	NA
	Amikacin	⩽8, >16	⩽8 to >32	32	>32	17·7
	Gentamicin	⩽2, >4	⩽2 to >8	4	>8	37·3
	Tobramycin	⩽2, >4	0·5 to >32	32	>32	6·7
	Imipenem	⩽2, >8	⩽1 to >8	>8	>8	24·7
	Meropenem	⩽2, >8	⩽1 to >8	>8	>8	30·3
	Doripenem	⩽1, >4	0·032 to >32	8	>32	18·3
	Piperacillin-Tazobactam	⩽8, >16	⩽4/4 to >64/4	>64/4	>64/4	3·7
	Ciprofloxacin	⩽0·5, >1	⩽0·5 to >2	>2	>2	10·0
	Fosfomycin w/G6P	⩽32, >32	⩽16 to >512	⩽16	128	56·0
	Colistin	⩽2, >2	⩽1 to >2	⩽1	>2	77·7
	Tigecycline	⩽1, >2	0·125 to 16	2	4	32·0
*Klebsiella pneumoniae*	Plazomicin	NA	⩽0·5 to 4	1	2	NA
	Amikacin	⩽8, >16	⩽8 to >32	32	>32	10·8
	Gentamicin	⩽2, >4	⩽2 to >8	4	>8	34·0
	Tobramycin	⩽2, >4	0·5 to >32	32	>32	3·7
	Imipenem	⩽⩽2, >8	⩽1 to >8	>8	>8	21·6
	Meropenem	⩽2, >8	⩽1 to >8	>8	>8	23·2
	Doripenem	⩽1, >4	0·032 to >32	8	>32	12·9
	Piperacillin to Tazobactam	⩽8, >16	⩽4/4 to >64/4	>64/4	>64/4	0·4
	Ciprofloxacin	⩽0·5, >1	⩽0·5 to >2	>2	>2	4·6
	Fosfomycin w/G6P	⩽32, >32	⩽16 to >512	⩽16	256	53·5
	Colistin	⩽2, >2	⩽1 to >2	⩽1	>2	73·0
	Tigecycline	⩽1, >2	0·125 to 16	2	4	25·7
*Escherichia coli*	Plazomicin	NA	⩽0·25 to 2	1	2	NA
	Amikacin	⩽8, >16	⩽⩽8 to >32	⩽8	>32	54·5
	Gentamicin	⩽2, >4	⩽2 to >8	8	>8	30·3
	Tobramycin	⩽2, >4	1 to >32	32	>32	21·2
	Imipenem	⩽2, >8	⩽1 to >8	⩽1	>8	54·4
	Meropenem	⩽2, >8	⩽1 to >8	⩽1	>8	78·8
	Doripenem	⩽1, >4	0·032 to >32	0·5	16	60·6
	Piperacillin-Tazobactam	⩽8, >16	⩽4/4 to >64/4	>64/4	>64/4	30·3
	Ciprofloxacin	⩽0·5, >1	⩽0·5 to >2	>2	>2	30·3
	Fosfomycin w/G6P	⩽32, >32	⩽16–256	⩽16	32	84·8
	Colistin	⩽2, >2	⩽1 to 2	⩽1	⩽1	100
	Tigecycline	⩽1, >2	0·25 to 16	1	2	69·7
*Enterobacter* spp	Plazomicin	NA	⩽0·5 to 2	1	1	NA
	Amikacin	⩽8, >16	⩽8 to >32	16	>32	34·6
	Gentamicin	⩽⩽2, >4	⩽2 to >8	2	4	76·9
	Tobramycin	⩽2, >4	1 to >32	32	32	15·3
	Imipenem	⩽2, >8	⩽1 to >8	>8	>8	15·4
	Meropenem	⩽2, >8	⩽1 to >8	>8	>8	34·6
	Doripenem	⩽1, >4	0·032 to >32	32	>32	15·4
	Piperacillin-Tazobactam	⩽8, >16	⩽4/4 to >64/4	>64/4	>64/4	0·0
	Ciprofloxacin	⩽0·5, >1	⩽0·5 to >2	2	>2	34·6
	Fosfomycin w/G6P	⩽32, >32	⩽16 to 256	32	128	42·3
	Colistin	⩽2, >2	⩽1 to >2	⩽1	⩽1	92·3
	Tigecycline	⩽1, >2	0·125 to 8	2	4	42·3

Isolates were highly resistant to tobramycin with only 6·7% of the strains being susceptible. Amikacin was active against 17·7% and gentamicin against 37·3% of the isolates. The vast majority (*n* =  242, 80·7%) of the isolates tested was non-susceptible to both amikacin and tobramycin, whereas 167 (55·7%) of them were resistant or intermediately susceptible also to gentamicin.

Plazomicin had an MIC range of ⩽0·25 to 4 μg/ml, with an MIC_50_ of 1 and an MIC_90_ of 2 μg/ml that were substantially lower than those for comparator aminoglycosides. Only thirteen *K. pneumoniae* isolates (4·3%) exhibited MICs of 4 μg/ml. These included one pandrug-resistant, 10 extensively drug-resistant and two MDR isolates. Those were resistant to tobramycin and amikacin (one was intermediate), whereas five were resistant or intermediate to gentamicin. None of those isolates was found to carry ribosomal methylases, while 7/13 possessed aminoglycoside modifying enzymes. In four of those isolates *aac(6*′*)-Ib* was the only gene detected, while *aac(3*′*)-Ia* and *aac(3*′*)-IIa* were revealed in one and two isolates respectively. No clear correlation between the MICs of plazomicin and the presence of aminoglycoside-modifying enzymes was observed.

Plazomicin MICs against a subset of enterobacterial isolates exhibiting specific phenotypic characteristics are listed in Table 2. Enterobacterial isolates with plazomicin MICs⩾8 μg/ml are not common. Landman has reported two *E. coli* isolates of the same ribotype, from two separate hospitals, in which genes for ribosomal methylases were not detected and the efflux pump inhibitor phenyl-arginine-beta-naphthylamide had no appreciable effect on the plazomicin MICs.^13^ Also 16 isolates with the New Delhi (NDM-1) MBL also producing 16S (rRNA) methylases exhibited high MICs (⩾8 μg/ml) to plazomicin.^4^

**Table 2:** Activities of plazomicin against 300 MDR enterobacterial isolates with different resistance phenotypes

Species	Phenotype	No. of isolates	MIC (μg/ml)						
			0·25	0·5	1	2	4	8	16
*Klebsiella pneumoniae*	KPC	25		7	12	5	1		
	ESBL, KPC	113		15	69	26	3		
	VIM	32		4	14	10	4		
	ESBL, VIM	43		8	22	9	4		
	KPC, VIM	10			7	3			
	ESBL, KPC, VIM	4			3	1			
	ESBL	14			9	4	1		
	Total	241		34	136	58	13		
*Escherichia coli*	KPC	9		3	4	2			
	VIM	5		2	3				
	ESBL, VIM	4	1		3				
	ESBL	15		3	8	4			
	Total	33	1	8	18	6			
*Enterobacter aerogenes*	KPC	1			1				
	ESBL, KPC	1		1					
	VIM	5			4	1			
	Total	**7**		1	5	1			
*Enterobacter cloacae*	KPC	2		1	1				
	VIM	15		8	6	1			
	KPC, VIM	1							
	ESBL, VIM	1			1				
	Total	19		10	8	1			

The low prevalence of 16S rRNA methylases in Enterobacteriaceae that has already been reported,^14^ as well as the absence of isolates producing the NDM-1 in Greece,^15^ is most probably the reason why we found no isolates with MICs>4 μg/ml.

The next-generation aminoglycoside plazomicin retains activity against all isolates of *K. pneumoniae, E. coli*, and *Enterobacter* spp. tested, including those with ESBL, KPC, and VIM-MBL resistance mechanisms and may represent a promising alternative for the treatment of MDR pathogens.

## References

[1] Armstrong ES, Miller GH (2010). Combating evolution with intelligent design: the neoglycoside ACHN-490.. Curr Opin Microbiol..

[2] Reyes N, Aggen JB, Kostrub CF (2011). *In vivo* efficacy of the novel aminoglycoside ACHN-490 in murine infection models.. Antimicrob Agents Chemother..

[3] Endimiani A, Hujer KM, Hujer AM, Armstrong ES, Choudhary Y, Aggen JB (2010). ACHN-490, a neoglycoside with potent *in vitro* activity against multidrug-resistant *Klebsiella pneumoniae* isolates.. Antimicrob Agents Chemother..

[4] Livermore DM, Mushtaq S, Warner M, Zhang JC, Maharjan S, Doumith M (2011). Activity of aminoglycosides, including ACHN-490, against carbapenem-resistant Enterobacteriaceae isolates.. J Antimicrob Chemother..

[5] Cass RT, Brooks CD, Havrilla NA, Tack KJ, Borin MT, Young D (2011). Pharmacokinetics and safety of single and multiple doses of ACHN-490 injection administered intravenously in healthy subjects.. Antimicrob Agents Chemother..

[6] Tsakris A, Poulou A, Pournaras S, Voulgari E, Vrioni G, Themeli-Digalaki K (2010). A simple phenotypic method for the differentiation of metallo-beta-lactamases and class A KPC carbapenemases in Enterobacteriaceae clinical isolates.. J Antimicrob Chemother..

[7] Clinical and Laboratory Standards Institute (2011). M100-S21, Performance standards for antimicrobial susceptibility testing; twenty first informational supplement.

[8] Tsakris A, Poulou A, Themeli-Digalaki K, Voulgari E, Pittaras T, Sofianou D (2009). Use of boronic acid disk tests to detect extended-spectrum *β*-lactamases in clinical isolates of KPC carbapenemase-possessing Enterobacteriaceae.. J Clin Microbiol..

[9] Doi Y, Arakawa Y (2007). 16S Ribosomal RNA Methylation: Emerging Resistance Mechanism against Aminoglycosides.. Clin Infect Dis..

[10] Wachino J, Shibayama K, Kurokawa H, Kimura K, Yamane K, Suzuki S (2007). Novel plasmid-mediated 16S rRNA m1A1408 methyltransferase, NpmA, found in a clinically isolated *Escherichia coli* strain resistant to structurally diverse aminoglycosides.. Antimicrob Agents Chemother..

[11] Aggen JB, Armstrong ES, Goldblum AA, Dozzo P, Linsell MS, Gliedt MJ (2010). Synthesis and spectrum of the neoglycoside ACHN-490.. Antimicrob Agents Chemother..

[12] Magiorakos AP, Srinivasan A, Carey RB, Carmeli Y, Falagas ME, Giske CG (2012). Multidrug-resistant, extensively drug-resistant and pandrug-resistant bacteria: an international expert proposal for interim standard definitions for acquired resistance.. Clin Microbiol Infect..

[13] Landman D, Babu E, Shah N, Kelly P, Bäcker M, Bratu S (2010). Activity of a novel aminoglycoside, ACHN-490, against clinical isolates of *Escherichia coli* and *Klebsiella pneumoniae* from New York City.. J Antimicrob Chemother..

[14] Galani I, Souli M, Panagea T, Poulakou G, Kanellakopoulou K, Giamarellou H (2012). Prevalence of 16S rRNA methylase genes in Enterobacteriaceae isolates from a Greek University hospital.. Clin Microbiol Infect..

[15] Grundmann H, Livermore DM, Giske CG, Canton R, Rossolini GM, Campos J (2010). Carbapenem-non-susceptible Enterobacteriaceae in Europe: conclusions from a meeting of national experts.. Euro Surveill.

